# Renoprotective Effects of AVE0991, a Nonpeptide Mas Receptor Agonist, in Experimental Acute Renal Injury

**DOI:** 10.1155/2012/808726

**Published:** 2012-01-29

**Authors:** Lívia Corrêa Barroso, Kátia Daniela Silveira, Cristiano Xavier Lima, Valdinéria Borges, Michael Bader, Milene Rachid, Robson Augusto Souza Santos, Danielle Gloria Souza, Ana Cristina Simões e Silva, Mauro Martins Teixeira

**Affiliations:** ^1^Laboratório de Imunofarmacologia, Departamento de Bioquímica e Imunologia, Instituto de Ciências Biológicas, UFMG, 31270-901 Belo Horizonte, MG, Brazil; ^2^Departamento de Fisiologia e Biofísica, Instituto de Ciências Biológicas, UFMG, 31270-901 Belo Horizonte, MG, Brazil; ^3^Departamento de Cirurgia, Faculdade de Medicina, UFMG, 30130-100 Belo Horizonte, MG, Brazil; ^4^Max Delbrück Center for Molecular Medicine, Berlin Buch, 13092 Berlin, Germany; ^5^Departamento de Patologia Geral, Instituto de Ciências Biológicas, UFMG, 31270-901 Belo Horizonte, MG, Brazil; ^6^Departamento de Microbiologia, Instituto de Ciências Biológicas, UFMG, 31270-901 Belo Horizonte, MG, Brazil; ^7^Departamento de Pediatria, Faculdade de Medicina, UFMG, 30130-100 Belo Horizonte, MG, Brazil

## Abstract

Renal ischemia and reperfusion (I/R) is the major cause of acute kidney injury in hospitalized patients. Mechanisms underlying reperfusion-associated injury include recruitment and activation of leukocytes and release of inflammatory mediators. In this study, we investigated the renal effects of acute administration of AVE0991, an agonist of Mas, the angiotensin-(1–7) receptor, the angiotensin-(1–7) receptor, in a murine model of renal I/R. Male C57BL/6 wild-type or Mas^−/−^ mice were subjected to 30 min of bilateral ischemia and 24 h of reperfusion. Administration of AVE0991 promoted renoprotective effects, as seen by improvement of function, decreased tissue injury, prevention of local and remote leucocyte infiltration, and release of the chemokine, CXCL1. I/R injury was similar in WT and Mas^−/−^ mice, suggesting that endogenous activation of this receptor does not control renal damage under baseline conditions. In conclusion, pharmacological interventions using Mas receptor agonists may represent a therapeutic opportunity for the treatment of renal I/R injury.

## 1. Introduction

Acute kidney injury (AKI) is defined as a rapid decrease of the glomerular filtration rate that occurs in minutes or days.Renal ischemia and reperfusion (I/R) is a major cause of AKI in hospitalized patients [1–3].Renal I/R triggers an inflammatory cascade clearly involved in the pathogenesis of AKI [4–6]. The control of inflammatory responses emerges as a putative therapeutic target to halt AKI [7]. Many regulatory systems modulate inflammation in renal tissue [4]. Among these systems, the Renin Angiotensin System (RAS) seems to exert a pivotal action in this scenario [8]. RAS is now regarded as a dual system with two opposite arms: the classical one formed by angiotensin converting enzyme (ACE), Angiotensin II (Ang II), and receptor type 1 (AT_1_) and the counter-regulatory arm comprising the enzyme ACE2, Angiotensin-(1–7) [Ang-(1–7)], and Mas receptor [9–13].

 Besides opposing AT_1_ receptor [14], the activation of Mas receptor by Ang-(1–7) or Ang-(1–7) agonists elicits renal effects [15–18]. Concerning experimental AKI, our team has recently shown that renal levels of Ang-(1–7) and ACE2 were significantly reduced whereas Mas receptor expression was increased [19]. However, our study was not able to clarify the precise function of the RAS at the early stages of renal I/R. In addition, data on the endogenous relevance of Mas receptor activation in renal tissue are still controversial [20, 21]. While Pinheiro et al. [20] showed that the genetic deletion of Mas receptor in C57Bl/6 mice led to glomerular hyperfiltration, proteinuria, and renal fibrosis, Esteban et al. [21] reported, otherwise, that renal deficiency of Mas diminished renal damage in unilateral ureteral obstruction and in I/R. Infusion of Ang-(1–7) to wild-type mice appeared to worsen the inflammatory response [21].

 More recently, we have shown that administration of Ang-(1–7) or the orally active agonist, the non-peptide compound AVE0991, exerted anti-inflammatory effects in experimental models of arthritis [19] and of nephritic syndrome [22]. Therefore, the purpose of the present study was to evaluate the renal effects of AVE0991 administration in a mice model of AKI, induced by renal ischemia following reperfusion.

## 2. Materials and Methods

### 2.1. Animals

Eight- to 10-week-old C57BL/6 wild-type male mice (Mas^+/+^) weighing 20–25 g or mice with genetic deletion of Mas receptor (Mas^−/−^) were obtained from the animal facility of the Universidade Federal de Minas Gerais. Animals were maintained under temperature-controlled conditions with an artificial 12-h light/dark cycle and were allowed standard chow and water ad libitum. The study was approved by the Ethics Committee of our Institution.

### 2.2. Renal Ischemia/Reperfusion Injury (I/R)

Mice were anesthetized with ketamine and xylazine (150 mg/kg and 10 mg/kg, resp.). Abdominal incision was performed to expose both renal pedicles. Ischemia was induced by totally occluding the renal pedicle for 30 minutes, using microsurgical clamps. After inspection for signs of ischemia, the wound was covered with cotton soaked with sterile PBS. After 30 min, clamps were released and blood-flow was reestablished, as confirmed by visual inspection of the kidneys. The wound was sutured in two layers using 5.0 sutures (Procare, Brazil). Throughout the experiments, body temperature was kept at 36–38°C by placing mice on a heating pad. Sham-operated animals were used as controls in all experimental protocols. These animals were also anesthetized and subjected to abdominal incision, exposing, and superficial manipulation of the renal pedicles. After recovery from anesthesia, mice were accommodated in individual metabolic cages (Tecniplast, Italy) for evaluation of renal function parameters. Urine was collected for the first 24 h after which mice were killed. Samples of plasma, renal, and lung tissues were collected and stored at −20°C for posterior analysis.

### 2.3. Study Protocol

The first set of experiments was to evaluate the effect of AVE0991 treatment in renal I/R injury. Therefore, wild type (Mas^+/+^) mice submitted to I/R were treated with subcutaneous (sc) injection (200 *μ*L) of AVE0991 (9.0 mg/kg) (Aventis Pharma Deutschland, Frankfurt, Germany), AVE group; or vehicle (10 mM KOH in 0.9% NaCl), VE group, immediately following 30 minutes of the renal ischemia and 12 h after renal reperfusion (AVE group) or vehicle (VE group). As control group, sham-operated Mas^+/+^ mice were also treated with vehicle (CT group).

The second set of the experiments had the aim to verify the endogenous role of Mas receptor in modulating experimental AKI. Thus, mice with genetic deletion of Mas receptor were submitted to the same protocol of renal I/R.

### 2.4. Determination of the MPO Activity

The extent of neutrophil accumulation in the kidney and lung tissue was measured by assaying myeloperoxidase (MPO) activity, as described previously [22]. Briefly, a portion of the kidney or lungs was removed and frozen in liquid nitrogen, homogenates were prepared in 1 mL of PBS containing 0.5% hexadecyltrimethyl ammonium bromide (HTAB) and 5 mM EDTA using a Dispomix tissue homogenizer (Medic Tools), and the protocol was followed as already described. Neutrophil number in each sample was calculated from a standard curve obtained from the peritoneal cavity after stimulation with 5% casein. The results were expressed as relative unit.

### 2.5. Differential Blood Cell Count

The total number of leukocytes was counted in a Neubauer chamber after staining with Turk's solution, and differential leukocyte counts were obtained after staining with May-Grunwald-Giemsa using standard morphologic criteria.

### 2.6. Assessment of CXCL1 in Serum

Levels of the chemokine CXCL1/KC were measured in serum using a commercial available enzyme-linked immunosorbent assay (ELISA) in accordance with the procedures supplied by the manufacturer (R&D Systems, Minneapolis, MN), as previously described by Souza et al. [14]. Results were expressed as picograms of cytokine per mL of serum.

### 2.7. Renal Function

To evaluate the effects of renal ischemia and reperfusion, as well as of the treatment with AVE0991 on renal physiology, several parameters were evaluated. First, mice were housed individually in metabolic cages (Tecniplast, Italy) three days before I/R procedure to adapt to the cages. I/R was then performed and animals were placed in the metabolic cages soon after recovering from anesthesia. Groups of animals were killed 24 h after reperfusion.

 At the end, 24 h urine samples were collected and centrifuged at 3,000 g for 5 min. Urine was used to determine osmolality and creatinine concentrations. Blood samples were collected from the lower abdominal cava vein, under ketamine and xylazine anesthesia (150 mg/kg and 10 mg/kg, resp.), and centrifuged at 2,000 ×g for 15 min at 4°C. The resulting plasma was used to measure osmolality and creatinine concentrations. Samples of urine and plasma were stored at −20°C until assessments.

 Creatinine concentrations were determined using an enzymatic kit (Bioclin/Quibasa, Belo Horizonte, MG, Brazil), and osmolality was determined by a freezing-point osmometer (microOsmetter, Calumet City, IL, USA). Osmolar clearance and free-water clearance were calculated. Finally, body weights were recorded daily.

### 2.8. Renal Histopathology

Paraffin-embedded sections (4 mm thick) were deparaffinized with xylene and rehydrated through a descending ethanol gradient. Histological sections were examined following H&E staining, and classified according to published standards [15, 16]. The degree of segmental glomerulosclerosis (glomerulosclerosis and nephron damage) was assessed by computer-aided image analysis of H&E-stained kidney sections. A semiquantitative score (glomerular and tubular injury index) was used to evaluate the degree of scarring as previously described [15, 16]. All scoring was performed in a blinded manner. The damage was scored semiquantitatively on a scale of 1 to 5.

### 2.9. Statistical Analysis

All results are presented as the mean ± SEM. Normalized data were analyzed by one-way ANOVA, and differences between groups were assessed using Student-Newman-Keuls post-test. The level of significance was set at *P* < 0.05.

## 3. Results and Discussion

It is well known that the classical RAS axis, composed of ACE/Ang II/AT_1_ receptor, plays a role in mediating AKI [17, 18, 23]. Recently, studies have also demonstrated an important role of the counterregulatory RAS axis, ACE2/Ang-(1–7)/Mas receptor, in several renal disorders, including AKI [19, 21, 24–28]. However, there are inconsistencies on the role of Mas receptor activation in renal disorders [20, 21]. As example, Pinheiro et al. [20] showed that genetic deletion of Mas receptor in C57BL/6 mice led to glomerular hyperfiltration, proteinuria, and renal fibrosis. In contrast, Esteban et al. [21] reported that renal deficiency for Mas diminished renal damage in unilateral ureteral obstruction and ischemia/reperfusion injury and the infusion of Ang-(1–7) to wild-type mice elicited inflammatory response. Therefore, whether the actions of Ang-(1–7) on renal function do indeed counter act those of Ang II in the context of disease states remains to be shown [20, 21]. In the present study, we investigated the renal effects of acute administration of the Mas receptor agonist, AVE0991, in a murine model of AKI caused by reperfusion of ischemic kidneys. Our major findings can be summarized as follows: (1) treatment with AVE0991 improves renal function and attenuates renal tissue damage; (2) the compound reduces infiltration of leukocytes in the kidney and overall tissue inflammation. On the other hand, despite the effectiveness the agonist of Mas receptor, AVE0991, in protecting the kidney against I/R injury, the same degree of renal damage was obtained in Mas^−/−^ mice submitted to experimental AKI. This finding suggests that there is no protective role for endogenous Mas activation. 

### 3.1. Renal Function and Inflammatory Parameters

As displayed in Table 1, bilateral ischemia (30 minutes) followed by reperfusion (24 h) causes an acute decrease of glomerular filtration characterized by the accumulation of serum creatinine (Table 1). Serum and urinary osmolality were also altered by I/R injury. The osmolality was elevated in the serum and reduced in the urine of mice submitted to I/R in comparison to sham-operated animals. The increase in serum osmolality can be attributable to the failure of the injured kidney to excrete nitrogen waste products and other osmotic active molecules, while the reduction in urinary osmolality probably reflects the loss of urinary concentration ability by ischemic renal tubules. Taken together, these functional data confirm the acute and significant impairment in renal function as a result of I/R injury [1, 2].

 I/R of the kidney is followed by a robust inflammatory reaction in the renal tubulointerstitium [29]. Studies in rodents have suggested that many components of the innate immune system contribute to renal injury after I/R, including neutrophils [30] and monocytes [31, 32]. Inflammatory cytokines and chemokines are generated in the ischemic kidney [30, 33] and likely orchestrate this broad inflammatory response. CXCL1/KC is a chemokine that promotes the recruitment and activation of inflammatory cells into renal tissue during I/R process [7]. In addition to renal damage, it was previously shown that extensive I/R, such as that of the renal or intestinal vascular beds, may be accompanied by remote organ (lung) and systemic inflammation [34, 35]. In the present study, we demonstrated an important increase in renal CXCL1/KC levels (Table 1). There was an increase in systemic and local (in renal tissue) accumulation of neutrophils, as detected by myeloperoxidade assay, which is in agreement with other studies [31, 35]. Pulskens et al. [36] and Roelofs et al. [37] found that neutrophils are the first inflammatory cells infiltrating the damaged kidney after reperfusion injury and there is much evidence suggesting that neutrophils are crucial for the development of reperfusion injury [37–39].

### 3.2. Effect of the Mas Receptor Agonist, AVE0991, in Renal I/R

**Figure 1:**
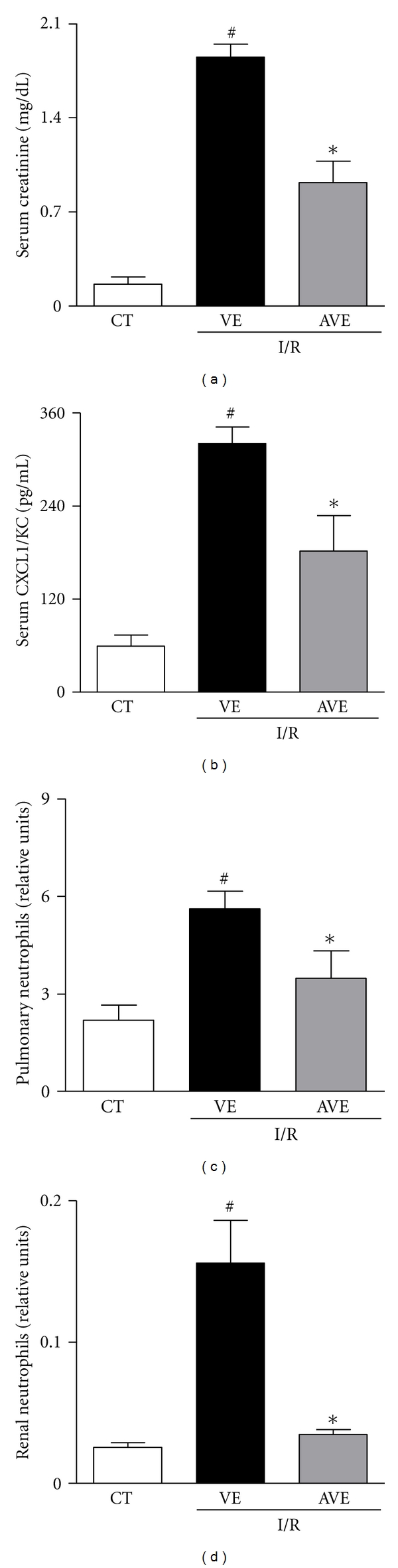
Effects of the treatment with AVE0991 in a model of renal ischemia and reperfusion in mice. AVE0991 (AVE, 9.0 mg/kg) or vehicle (VE; 10 mM KOH in 0.9% NaCl) was given immediately after ischemia and 12 h after reperfusion. All analyses were performed at 24 h after reperfusion. Serum creatinine (a), number of neutrophils in blood (b), levels of CXCL1/KC (c), and relative number of neutrophils in renal tissue (d) are shown as the mean ± SEM from six mice per group. *P < 0.05 when compared with VE-treated animals; ^#^ *P* < 0.05 when compared with sham-operated animals (CT, control).

Recent studies have evidenced a renoprotective and anti-inflammatory effect of ACE2/Ang-(1–7)/Mas receptor axis activation in the context of numerous disease models [26, 40]. These findings encouraged us to investigate the effect of an agonist of Mas receptor, AVE0991, in experimental AKI. The elevation of serum creatinine is one of the most important biomarkers of impaired glomerular filtration present in AKI [41]. We demonstrated that AVE0991 was able to attenuate the increase of serum creatinine, when compared to the vehicle-treated group (Figure 1(a)).

 There was a marked infiltration of neutrophils in renal and pulmonary tissues after I/R injury (Figures 1(b)-1(c)). Treatment with AVE0991 significantly decreased neutrophil influx in both the kidney and lungs (Figures 1(b) and 1(c)). Decrease in neutrophil accumulation in both organs was associated with reduced production of CXCL1/KC, as seen by lower circulating levels of this chemokine in AVE-treated animals than in vehicle group (Figure 1(d)). Systemic and remote organ inflammation frequently accompanies severe I/R injury [34, 42] and may contribute to the fatal outcome observed in reperfused mice. The lung is the most commonly affected remote organ during systemic inflammation [34, 35, 43, 44]. Therefore, protection against systemic inflammation may have accounted for the overall beneficial effects of AVE0991 treatment. We have recently shown that Mas receptor activation decreased neutrophil migration and accumulation in models of arthritis [45], suggesting the overall capacity of Mas receptor activation in controlling the influx of these cells during acute inflammatory response.

**Figure 2:**
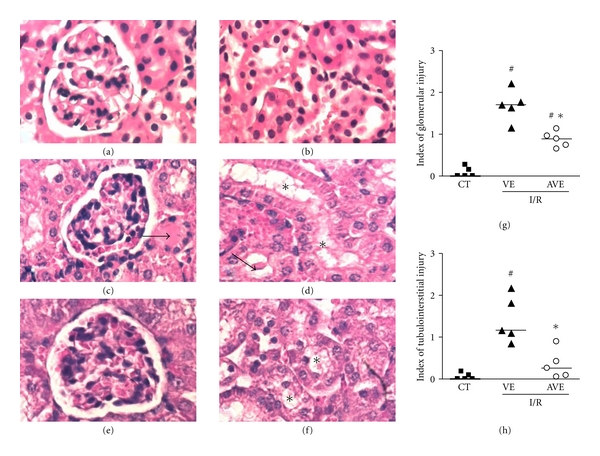
Effects of the treatment with AVE0991 in histological injury in a model of renal ischemia and reperfusion in mice. AVE0991 (AVE, 9.0 mg/kg) or vehicle (VE; 10 mM KOH in 0.9% NaCl) was given immediately after ischemia and 12 h after reperfusion. All analyses were performed at 24 h after reperfusion. Representative photomicrographs show H&E-stained sections from sham-operated animals (CT, a, b) and animals subjected to I/R, which were treated with vehicle (VE, c, d) and AVE0991 (AVE, 9 mg/kg, e, f). There was severe renal damage with vacuolization of tubular epithelium (c) (insert) and tubular dilation and protein casts (arrow) and extensive tubular necrosis (d) in mice subjected to I/R. Original magnification: ×600. Index of glomerular injury (g) and index of tubulointerstitial injury (h) were graded in a blind manner, as described in Material and Methods. Symbols represent results in single animals, and the trace is median value for all animals. *P < 0.05 when compared with VE-treated group; ^#^ *P* < 0.05 when compared with CT.

 As an attempt to quantify the protective action of AVE0991 at the level of the renal tissue, the degree of tubular and glomerular injuries was evaluated in mice subjected to I/R. As expected, sham-operated animals (control group) had well-preserved glomerular and tubular architecture (Figures 2(a)-2(b)). Thirty minutes of ischemia followed by 24 h of reperfusion caused focal acute tubular necrosis, intense tubular vacuolization in proximal tubules (asterisks), cast formation (arrows), and tubular dilatation as observed in vehicle-treated animals submitted to I/R (Figures 2(c)-2(d)). On the other hand, the administration of AVE0991 improved renal damage in animals submitted to I/R (Figures 2(e)-2(f)). The renal tissue of AVE-treated animals had a significant decrease in the indexes of renal injury at both glomerular and tubular sites when compared to vehicle group (Figure 2(h)).

 Studies evaluating the effect of Ang-(1–7) or AVE0991 in models of chronic kidney disease have shown that these molecules might be renoprotective [25, 26]. In this regard, Zhang et al. showed that Ang-(1–7) infusion ameliorates glomerular sclerosis in experimental glomerulonephritis [24]. In addition, the beneficial effect of ACE2 overexpression in experimental diabetic nephropathy may also be due to the increase of Ang-(1–7) levels [24]. These studies concur with our findings that the agonist of Mas receptor, AVE0991, attenuates renal damage in this model of I/R injury. Based on our previous results with experimental arthritis [45], we believe that AVE0991 exerted renoprotective actions mainly through Mas receptor activation. To corroborate this idea, our group has previous shown that the mRNA expression for Mas receptor is increased in I/R model [19]. However, we cannot rule out the possibility that other mechanisms could contribute to the beneficial effects of AVE0991. In this regard, AVE0991 could interact with ACE by inhibiting the renal activity of this enzyme.

**Figure 3:**
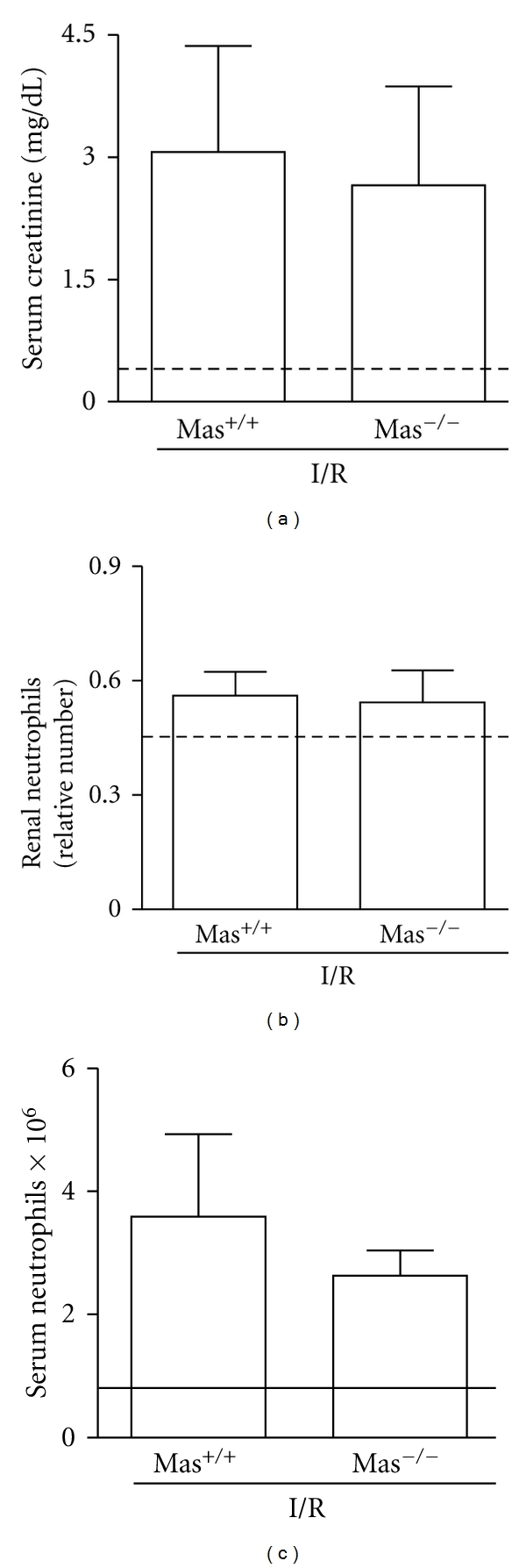
Ischemia and reperfusion in mice with genetic deletion for the Ang-(1–7) Mas receptor (Mas^−/−^) or WT mice (Mas ^+/+^). Animals were subjected to 30 min of ischemia followed by 24 h of reperfusion. Serum creatinine (a), relative number of neutrophils in renal tissue (b), and number of neutrophils in blood (c) are shown as the mean ± SEM of six mice per group.

To verify the endogenous role of Mas receptor activation in the pathogenesis of renal I/R, we induced AKI in mice with genetic deletion of this receptor (Mas^−/−^). As demonstrated in Figure 3, the absence of Mas receptor did not change the degree of renal function impairment, as assessed by serum creatinine concentration (Figure 3(a)). Moreover, there was no change in inflammatory response, as measured by the number of systemic and renal neutrophils (Figures 3(b)-3(c)). The increase of Mas receptor could represent more a compensatory response to renal damage rather than an endogenous regulatory mechanism. In this regard, we have previously obtained the same finding in a model of arthritis induced by adjuvant [45]; that is, the phenotype of Mas-deficient mice was not major when compared to pharmacological administration of AVE0991, which were able to efficiently control articular inflammation through Mas receptor activation. Similarly, endogenous activation of Mas receptor does not appear to exert a major role in I/R model.

The present results are in contrast to those of Esteban et al. [21], which showed less intense reperfusion injury in Mas^−/−^ mice. These data are difficult to reconcile. However, there are substantial differences between the experimental protocols for producing I/R model and for the Mas receptor agonist administration such as which agonist was administered, AVE0991 or Ang-(1–7), the via of administration, and the dose used. For example, in the present study, we have used a model of bilateral I/R (30 min), while Esteban et al. performed unilateral I/R (25 min) with contralateral nephrectomy [21] that could at least in part account for the differences observed. Renal effects of Mas receptor agonists, Ang-(1–7) and AVE0991, appear to be importantly influenced by experimental conditions and the level of RAS activation. Despite these inconsistencies, our results corroborate emerging data showing that the major overall effects of ACE2/Ang-(1–7)/Mas receptor axis stimulation are anti-inflammatory [19, 21, 24–28]. As such, one would expect that absence of this pathway would be associated with no phenotype, as observed here, or even worsening of the inflammatory response [37].

 In conclusion, treatment with AVE0991, a nonpeptide Mas agonist, attenuated renal functional impairment, decreased the local and systemic inflammatory responses, and reduced glomerular and tubulointerstitial damage in a murine model of AKI induced by bilateral I/R injury. These results show that activation of Mas receptor is renoprotective, at least in part by anti-inflammatory actions, in mice subjected to AKI, a tenet that clearly deserves further investigation in the context of human disease.

## Figures and Tables

**Table 1:** Effect of renal ischemia and reperfusion on renal function parameters, local CXCL1 production, and local and remote neutrophil accumulation. Urinary creatinine (mg/dL), serum creatinine (mg/dL), serum osmolality (mOsm/Kg H_2_O), urinary osmolality (mOsm/Kg H_2_O), serum CXCL1 (pl/mL serum), and renal and pulmonary neutrophils (relative units).

	Control	I/R	*n*	*P*
Urinary creatinine (mg/dL)	700 ± 5.1	170 ± 1.2*	6	0.001
Serum creatinine (mg/dL)	0.29 ± 0.1	5.34 ± 1.2*	4	0.016
Serum osmolality (mOsm/Kg H_2_O)	317 ± 10.4	360 ± 17.8*	6	0.006
Urinary osmolality (mOsm/Kg H_2_O)	3996 ± 340	905 ± 69*	6	0.06
Serum CXCL1/KC (pg/mL)	94 ± 70	940 ± 164*	6	0.008
Renal neutrophils (relative units)	0.26 ± 0.13	5.3 ± 1.2*	4	0.016
Lung neutrophils (relative Units)	2.2 ± 0.5	5.6 ± 0.5*	5	0.0015
